# Longitudinal changes in epigenetic clocks predict survival in the InCHIANTI cohort

**DOI:** 10.1038/s43587-026-01066-6

**Published:** 2026-03-13

**Authors:** Pei-Lun Kuo, Ann Zenobia Moore, Toshiko Tanaka, Daniel W. Belsky, Ake Tzu-Hui Lu, Steve Horvath, Stefania Bandinelli, Luigi Ferrucci

**Affiliations:** 1 National Institute on Aging, National Institutes of Health, Baltimore, MD USA; 2 Robert N Butler Columbia Aging Center and Department of Epidemiology, Mailman School of Public Health, Columbia University, New York, NY USA; 3 Altos Labs, San Diego, San Diego, CA USA; 4 Department of Human Genetics, David Geffen School of Medicine, University of California Los Angeles, Los Angeles, CA USA; 5 Geriatric Unit, Local Health Unit Tuscany Centre, Firenze, Tuscany Italy

**Keywords:** Predictive markers, Molecular biology, Ageing

## Abstract

Epigenetic clocks derived from DNA methylation patterns are among the most promising biomarkers of biological aging^1–7^, as they capture molecular signatures that predict morbidity and mortality beyond chronological age. Although cross-sectional assessments of epigenetic age have been linked consistently to health outcomes and lifespan, it remains unclear whether the rate of change in these clocks over time provides additional insight into aging trajectories. In this longitudinal study of 699 adults from the InCHIANTI cohort followed for up to 24 years, we evaluated whether temporal acceleration of several epigenetic clocks—including first-, second- and third-generation epigenetic clocks—was associated with mortality. We found that faster increases in several clocks were linked robustly to higher risk of death, independent of baseline epigenetic age and other confounders. These findings suggest that dynamic changes in epigenetic aging reflect evolving health status and may serve as sensitive indicators for interventions aimed at extending healthspan and longevity.

## Abstract (web summary)

Epigenetic clocks are promising biomarkers of biological aging but require longitudinal evaluation. In this longitudinal study, the authors evaluated whether temporal acceleration of multiple epigenetic clocks was associated with mortality, and report that faster increases in several clocks were linked to higher risk of death, independent of baseline epigenetic age and other confounders.

## Main

The geroscience hypothesis posits that the biology of aging is the root cause of chronic diseases and functional impairments that emerge in late life^8–10^. Thus, slowing down the pace of biological aging may prevent or delay the onset of diseases and disabilities and prolong the healthspan, possibly expanding the proportion of life that people live in good health^8,9,11,12^. A first step in accomplishing this goal is to develop metrics of biological aging that can identify faster aging people and track the effect of interventions aimed at slowing down the aging process^8,11,13^.

Over the past years, several proxy biomarkers of biological aging have been developed and validated, with the most advanced using data from DNA methylation^14^. Broadly termed ‘epigenetic clocks,’ these methylation-based markers of aging have been shown to predict several adverse health outcomes, including mortality, independently of chronological age^15^. Early in the 2010s, the Hannum clock and Horvath clock were developed, combining information on percent methylation in selected DNA sites to obtain a score that best correlated with chronological age^1,2^. Second-generation clocks, such as DNAmPhenoAge, DNAmGrimAge and DNAmGrimAge v.2, were developed using mortality and other blood biomarkers^3,4,6^. The DunedinPOAm_38 and DunedinPACE were developed using the pace of biological aging estimated from a combination of longitudinal trends of several phenotypes in a birth cohort^5,7^. However, whether longitudinal changes in these phenotypes provide additional information on health outcome prediction over and beyond one single measure has not been demonstrated^11,16^. Based on cross-sectional studies, we cannot definitively exclude that deviations of DNA methylation age from chronological age are determined early in life and are not modulated by behavioral, environmental exposures or changes in health status^11,17,18^. In addition, if biological aging clocks are to be used to track the effectiveness of intervention over time, it is important to demonstrate that deviations of epigenetic clock trajectories reflect meaningful changes in health status^19^. In this study, we use longitudinal data collected in the InCHIANTI study to test the hypothesis that longitudinal changes in epigenetic clock predict mortality over and beyond the same epigenetic clock assessed at one point in time.

We studied 699 participants of the InCHIANTI study—a population-based study of factors affecting loss of mobility in late life performed in two towns close to Florence, Italy^20^. Among these 699 participants, 308 (44%) were male. Of the initial cohort, 396 participants died over the 24 years of follow-up (mortality rate: 29.14 deaths per 1,000 person-years). Median (range) of follow-up time was 21.5 (interquartile range 15.6–23.4) years. DNA methylation was measured using the Illumina Infinium HumanMethylation450 BeadChip^21^ and percentage of methylation ranged between 0 (completely unmethylated) and 1 (completely methylated) at each CpG site^22–24^. A total of 1,721 samples from 699 participants (376 participants measured at two timepoints, 323 participants measured at three timepoints) were used in the analysis. The Hannum clock, Horvath clock, DNAmPhenoAge and two versions of DNAmGrimAge were calculated through web-based calculator and their estimates were adjusted for age to obtain ‘epigenetic age acceleration’ metrics. We also calculated the DunedinPOAm_38 and DunedinPACE clocks that already estimate age acceleration.

The average baseline chronological age of the participants was 63 years (s.d. = 16), and the lowest mean value for epigenetic aging markers was 55.9 years (s.d. = 17.4) for DNAmPhenoAge, whereas the highest mean was 67.2 years (s.d. = 12.9) for DNAmGrimAge v.2 (Table 1). The mean values for DunedinPOAm_38 and DunedinPACE were 1.02 (s.d. = 0.09) and 1.06 (s.d. = 0.12). Across the other epigenetic markers of aging, the smallest mean annual rate of change was observed for DNAmGrimAge v.2, 0.66 (s.d. = 0.29), whereas DNAmPhenoAge had the largest value, 0.96 (s.d. = 0.50) (Table 1). The correlations between baseline epigenetic markers of aging and annual rates of change in epigenetic markers of aging ranged from −0.20 to −0.49 (Supplementary Fig. 1) and their correlation with chronological age and among themselves are summarized in Supplementary Fig. 2. The distribution of the slopes across different epigenetic clocks and their modest correlation with chronological age (range 0.01–0.18) are shown in Supplementary Fig. 3.

**Table 1:** Characteristics of participants in InCHIANTI (*n* = 699)

Characteristics	Mean (s.d.) or *n* (%)
Baseline chronological age	63.11 (15.68)
Sex	
Male	308 (44.06%)
Female	391 (55.94%)
Epigenetic markers of aging at baseline	
Hannum Clock	66.86 (16.01)
Horvath Clock	61.77 (14.18)
DNAmPhenoAge	55.87 (17.44)
DNAmGrimAge	62.58 (12.85)
DNAmGrimAge v.2	67.18 (12.85)
DunedinPOAm_38	1.02 (0.09)
DunedinPACE	1.06 (0.12)
Annual rate of longitudinal changes in epigenetic markers of aging	
Hannum Clock	0.80 (0.44)
Horvath Clock	0.78 (0.43)
DNAmPhenoAge	0.96 (0.50)
DNAmGrimAge	0.69 (0.28)
DNAmGrimAge-version 2	0.66 (0.29)
DunedinPOAm_38	0.0000061 (0.0078)
DunedinPACE	0.0044 (0.0085)

Seven epigenetic markers of aging were assessed, including Hannum clock, Horvath clock, DNAmPhenoAge, DNAmGrimAge, DNAmGrimAge v.2, DunedinPOAm_38, and DunedinPACE.

**Fig. 1:**
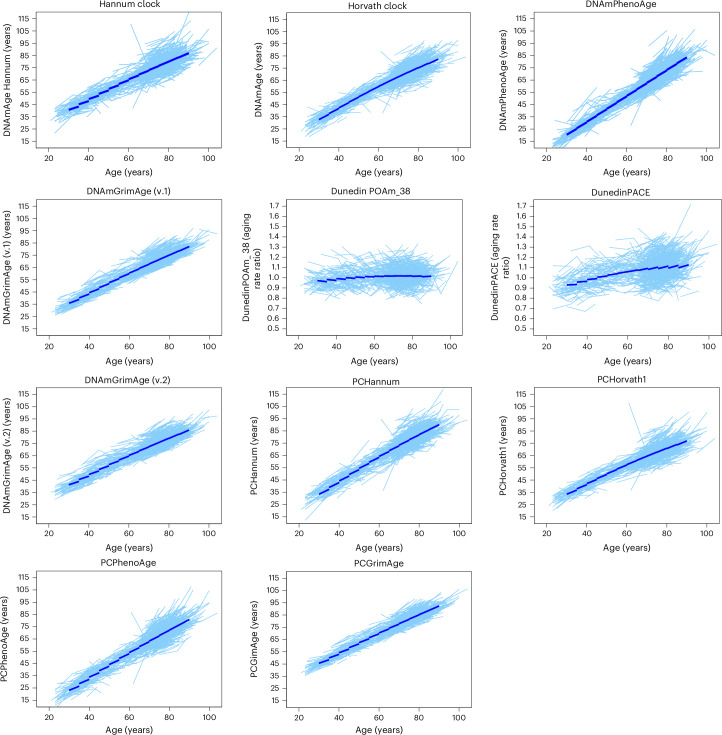
Spaghetti plots showing longitudinal trajectories of several epigenetic clocks. Individual (‘spaghetti’) plots showing longitudinal trajectories of epigenetic clock (light blue) estimates in 699 participants with repeated DNA methylation measures. Each line represents a person’s change over time, illustrating the variability in epigenetic aging rates. For selected clocks, longitudinal trajectories derived using the principal-components-based method are also displayed, demonstrating comparable patterns of change. The dark blue line represents the fitted trajectories across all ages. Source data

Figure 1 shows ‘spaghetti’ plots illustrating crude longitudinal changes in epigenetic clocks for 699 participants with repeated-measures data. The consistency of linear change over time within individual participants is striking, with only a few people showing substantial deviations. Some clocks seem to tick slower/faster than others. DunedinPOAm_38 and DunedinPACE already express pace of epigenetic aging, and therefore longitudinal trajectories over time should be interpreted as age-accelerations or deceleration. Consistent with previous reports^5,25^, DunedinPOAm_38 trajectories showed relative stable pace of epigenetic aging across different age groups, whereas the DunedinPACE pace of epigenetic age increased over time^5^. In addition, the rate of increase in DunedinPACE accelerated as participants got older (Supplementary Table 1). Estimated age-specific annual rates of change in epigenetic age for different epigenetic clocks are shown in Supplementary Table 1.

**Fig. 2:**
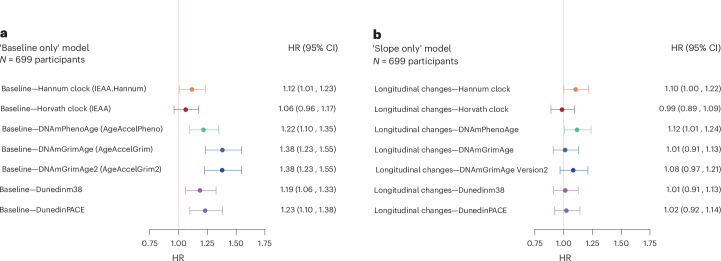
Forest plots showing relationship between baseline of and mortality and longitudinal changes in epigenetic clocks and mortality. **a**,**b**, HRs and 95% CI for mortality associated with baseline epigenetic clock values (**a**) and annual rates of longitudinal change in the same clocks (**b**). Both models were adjusted for chronological age, sex and study site. To aid comparison across clocks, all coefficients are standardized. Source data

**Fig. 3:**
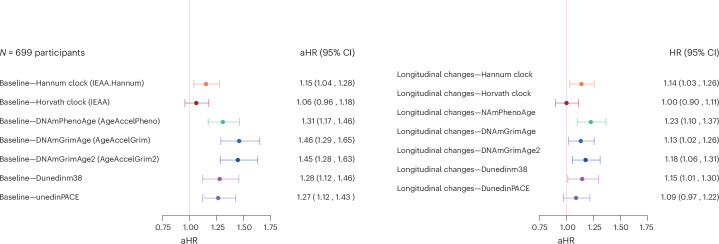
Forest plots showing relationship between baseline of and longitudinal changes in epigenetic clocks and mortality, when both of them are in the model. aHRs and 95% CI for mortality associated with baseline epigenetic clock values and change over time of the same epigenetic clock, adjusting for chronological age, sex and site. To aid comparison across clocks, all coefficients are standardized. Source data

The forest plots in Figs. 2 and 3 provide visual representations of the relationship between epigenetic clocks and mortality. In the ‘Baseline only’ model, all epigenetic markers of aging except for Horvath clock were associated with mortality (adjusted hazard ratio (aHR) (95% confidence interval (CI): 1.12 (1.01–1.23) for Hannum clock, 1.06 (0.96–1.17) for Horvath clock, 1.22 (1.10–1.35) for DNAmPhenoAge, 1.38 (1.23–1.55) for DNAmGrimAge, 1.38 (1.23–1.55) for DNAmGrimAge v.2, 1.19 (1.06–1.33) for DundedinPOAm_38 and 1.23 (1.10–1.38) for DunedinPACE). In the ‘Slope only’ model, only the longitudinal changes in Hannum clock and DNAmPhenoAge were associated with mortality (aHR (95% CI)): 1.10 (1.00–1.24) for Hannum clock, 0.99 (0.89–1.09) for Horvath clock, 1.12 (1.01–1.24) for DNAmPhenoAge, 1.01 (0.91–1.13) for DNAmGrimAge, 1.08 (0.97–1.21) for DNAmGrimAge v.2, 1.01 (0.91–1.13) for DundedinPOAm_38, and 1.02 (0.92–1.14) for DunedinPACE). When both baseline and longitudinal changes were included in the model (Fig. 3), the associations between baseline measures and mortality were generally stronger than the estimates from the ‘Baseline only’ model, and the associations between longitudinal changes and mortality were generally stronger than the estimates from the ‘Slope only’ model (Fig. 2). Results remained substantially unchanged after additional adjustment of Life Simple Seven (LS7) (Supplementary Fig. 4). When we repeated the analysis using epigenetic clocks reconstructed by principal components (PC clocks; epigenetic clocks calculated adopting technical noises reduction algorithms), the associations between changes in epigenetic clocks and mortality using PC clocks were very similar to those obtained using the original clocks, except for an observable improvement in Horvath Clock (Supplementary Fig. 5). We explored whether the epigenetic estimators of circulating proteins included in DNAmGrimAge v.2 were associated with mortality and found that longitudinal changes in methylation-based estimation GDF-15, Cystatin-C, TIMP-1 and logCRP were associated significantly with mortality (Supplementary Table 2). Furthermore, when we examined whether the relationship with mortality differs between the older and the younger group, we found that the association between baseline epigenetic ages (for Dunedinm38 and DunedinPACE) and mortality are weaker in older age group, whereas the relationship between longitudinal changes in epigenetic ages and mortality was similar between the younger group and the older group (Supplementary Fig. 6). Using age as a continuous variable in the same model did not substantially change the results (results not shown).

The performance of the different models for mortality prediction was assessed using a variety of measures, including C-statistics, integrated discrimination improvement index (IDI) and net reclassification index (NRI). The results are presented in Supplementary Tables 3 and 4. The models that included both baseline and longitudinal change for DNAmGrimAge v.2, DNAmGrimAge, DNAmPhenoAge and DunedinPACE had the highest C-statistics (Concordance index: 0.808 for DNAmGrimAge v.2, 0.806 for DNAmGrimAge, 0.801 for DNAmPhenoAge and 0.800 for DunedinPACE). The IDI and NRI also showed that the integration of baseline value and longitudinal changes in epigenetic clocks demonstrated improvement in mortality prediction performance (IDI: 0.017 (0.005–0.035) for DNAmPhenoAge, 0.027 (0.010–0.045) for DNAmGrimAge; NRI: 0.251 (0.084–0.320) for DNAmPhenoAge, 0.236 (0.053–0.316) for DNAmGrimAge), with finer comparisons limited by moderate sample size.

In this study, we found that, after adjustment for baseline epigenetic clock and independent of chronological age and sex, changes in most epigenetic clocks predict differential mortality. Second-generation clocks trained using mortality for reference (DNAmPhenoAge, DNAmGrimAge, DNAmGrimAge v.2) or third-generation clocks trained against longitudinal phenotypic changes (DunedinPOAm_38, DunedinPACE) performed better for mortality prediction than first-generation clocks, as evidenced by higher concordance indices and integrated discrimination improvement indices. Specifically, models fit with second-generation clocks (DNAmPhenoAge, DNAmGrimAge, DNAmGrimAge v.2) had the highest net reclassification indexes in our study cohort.

The association between baseline epigenetic clock and mortality was reported previously by many studies. Higgins-Chen and colleagues showed that DNAmPhenoAge and DNAmGrimAge had a stronger association with mortality than the Hannum clock and Horvath clock, even after improving their reliability using new computational techniques^19^. Similarly, Belsky and colleagues found that DunedinPOAm_38 and DunedinPACE were associated more strongly with mortality than Hannum clock and Horvath clock, consistent with our results^5,7^. By leveraging longitudinal data, we were able to include both baseline and longitudinal measurements of epigenetic clocks in our analyses and demonstrated that the mortality prediction was substantially higher when both baseline and longitudinal estimates of epigenetic aging were considered jointly. A puzzling result was that different epigenetic clocks showed different longitudinal slopes of epigenetic aging, all of which were lower than 1 epigenetic year per year of chronological age, which may result from regression to mean during the process of clock development, or indicate part of biological aging not captured by epigenetic changes. Although the reason for this finding is unclear, the longitudinal trajectories in epigenetic clocks found in our study are consistent with the findings from Swedish Adoption/Twin Study of Aging (SATSA) and Berlin Aging Study^25–28^. We further reported the estimated rate of changes in these clocks in Supplementary Table 1, and showed that the rate of changes in some clocks (GrimAge v.1 and v.2, DunedinPACE) accelerated with age. Although data before adulthood are unavailable in our cohort, Mulder et al. found that early exposures, including prenatal environment and lifestyle, contributed to the changes in the CpG sites used for generating epigenetic clocks^29^. In the future, developing epigenetic clocks estimated using repeated measures of methylation and correlating them with change in age-phenotypes may shed light on the biological meaning of this result.

When not adjusted for baseline values, longitudinal changes in the epigenetic clocks were associated with mortality only for the Hannum clock and DNAmPhenoAge, but not for the other clocks. These findings cannot be compared directly with the literature because data on longitudinal changes in epigenetic markers is scant^25,26^. One possible interpretation of these findings is that longitudinal trajectories of epigenetic clocks may be affected by ‘regression to the mean’ and including baseline values in the same equation may account, at least partially, for this effect. This is consistent with the moderate negative correlation between baseline measurement and longitudinal changes in epigenetic clocks found in the Berlin Aging Study^26^. Our results highlight the importance of integrating both the baseline and longitudinal changes of epigenetic clocks in the model^30^. Overall, we interpret our findings as demonstrating that epigenetic age is not a straight trajectory over time but shows deviations in response to meaningful changes in health, and that is why considering such deviation improves our mortality prediction. Indeed, recent studies have shown that both exposures in early life and lifestyles (such as smoking, medications for hypertension/diabetes) during adulthood are associated with methylation of CpG sites and changes in epigenetic clocks^27,29,31^. On the other hand, increased variability across CpG sites has been observed, and other studies have attempted to understand the roles of lifestyle variations (such as smoking, calorie restriction and alcohol drinking)^32–35^. Future research in larger cohorts and with longer follow-up should disentangle stochastic aging processes from deterministic influences of environment and lifestyle (for example, smoking, diet, sleep and physical activity) and may shed light on the mechanisms driving the epigenetic clock.

In our analysis, second-generation (DNAmPhenoAge, DNAmGrimAge, DNAmGrimAge v.2) and third-generation (DunedinPOAm_38, DunedinPACE) clocks were superior to the first-generation clocks (Hannum clock, Horvath clock) in mortality prediction. This was not unexpected since second-generation clocks were optimized to predict mortality and third-generation clocks built upon the longitudinal changes of many clinically important phenotypes. In contrast, first-generation clocks were trained on chronologic age, which obscure deviation from biological age that may convey important information on mortality. DNAmPhenoAge and DNAmGrimAge seemed to have better net reclassification ability than DunedinPOAm_38 and DunedinPACE. One plausible reason is that the optimization of DunedinPOAm_38 and DunedinPACE did not directly involve mortality information at all. Another plausible reason is that DNAmPhenoAge and DNAmGrimAge were trained on adults across a wider age range than DunedinPOAm_38 and DunedinPACE, which were trained on younger adults at 26–38 and 26–45 years, respectively, and their predictive validity may not fully extend to older ages^5,7^. It is also possible that DunedinPACE captures more deterministic components of aging than other clocks. This notion is also suggested by recent studies showing that (1) the first and second generations of clocks may be driven in large part by the accumulation of stochastic variation^32^, and (2) when temporal order between epigenetic aging and frailty—an important predictor of morality—were examined, only the change in DunedinPACE preceded changes in the frailty index^25^. Epigenetic plasticity is probably highest in early life, when the genome’s biological programs must adjust to the surrounding environment. With aging, this capacity for adaptation seems to decline, as has been proposed for many resilience mechanisms.

Our study demonstrates that longitudinal trajectories of epigenetic clocks can serve as reliable tools in observational studies to detect health changes driven by biological or environmental factors linked to mortality and in intervention trials to assess strategies that slow biological aging and reduce its detrimental consequences^10^. Although our study focused on mortality, it will be important to expand these observations to different outcomes, such as multimorbidity and disability, that are theoretically associated with accelerated aging^11,12^. Future research should also investigate whether interventions that slow the changes in epigenetic markers of aging can improve healthspan and life expectancy. Ultimately, it will be important to understand what biological mechanism drives the changes in the trajectories of epigenetic clocks and the phenotypes correlate with these changes.

There are several strengths and limitations in our study. All participants were of European ancestry. An important goal of future research is to develop epigenetic clocks from highly diverse populations and verify that their cross-sectional values and changes over time are predictive of health outcomes across races and sex. The lack of overlapping CpG sites among these epigenetic clocks and the poor correlation among them reported in the literature suggests that they may convey much more information than we can currently summarize. Thus, larger studies with the use of artificial intelligence are needed to unveil underlying epigenetic networks, to refine these tools and to facilitate their translation into clinical applications. Although cross-sectional differences in epigenetic age may reflect underlying genetic background present since birth, longitudinal changes are less likely to be affected by time-invariant confounders such as genetics, particularly in older people. A key future direction is therefore the development of epigenetic clocks calibrated to longitudinal changes in global health phenotypes, based on percent methylation changes over time. Our results provide evidence that longitudinal changes in epigenetic clock values reflect meaningful changes in health, setting the scene for expanding and translating this line of research.

## Methods

### Study design and participants

The InCHIANTI study participants were recruited from two Chianti regions of Tuscany, Italy (1998–2000). Details about this study have been described previously^20^. For the present analysis, we included 699 participants with DNA methylation measurements obtained at two or three timepoints (in the 1998, 2007 and 2013 study visits). InCHIANTI was approved by the Italian National Institute of Research and Care of Aging Institutional Review Board, and study participants signed informed consents. This study adheres to the Strengthening the Reporting of Observational Studies in Epidemiology (STROBE) reporting guideline^36^.

### Epigenetic markers of aging—epigenetic clocks

DNA methylation was measured using the Illumina Infinium HumanMethylation450 BeadChip^21^. Data quality control included background correction, multidimensional scaling and checking that self-reported sex matched the estimated pattern of sex chromosomes^23^. R packages ‘minfi’ and ‘SeSAMe’ were used for quality control and to generate methylation beta values, which represent the percentage of methylation ranging between 0 (completely unmethylated) and 1 (completely methylated) at each CpG site^22–24^. A total of 1,721 samples from 699 participants (376 participants measured at two timepoints, 323 participants measured at three timepoints) were used in the analysis.

We used DNA methylation data to calculate seven epigenetic clocks. The first-generation (Hannum clock, Horvath clock) and second-generation (DNAmPhenoAge and two versions of DNAmGrimAge) clocks were calculated through a web-based calculator (https://horvath.genetics.ucla.edu/html/dnamage/). Because these five epigenetic clocks have been found consistently associated with chronological age, their age-adjusted values are often considered as ‘epigenetic age acceleration’ (despite not being measured longitudinally). The third-generation epigenetic clocks (DunedinPOAm_38 and DunedinPACE) were originally developed to estimate the pace of biological aging and, therefore, are expressed on a different scale. These clocks were estimated by the DunedinPOAm_38 and DunedinPACE R packages (https://github.com/danbelsky/DunedinPoAm38 and https://github.com/danbelsky/DunedinPACE)^5,7^.

Annual rate of changes of epigenetic clocks for each person were estimated by linear regression analyses. Because different epigenetic clocks were derived differently and are expressed on a different scale, all the baseline level and longitudinal changes in epigenetic clocks were standardized to the unit of s.d. to allow fair comparison.

A recent literature proposed that PC clocks have better reliabilities than the original versions^19^. Therefore, we conducted the analysis using the PC versions of the clocks^19,37^. Furthermore, since DNAmGrimAge v.2 contained several protein proxies, we also reported the associations between these protein proxies and mortality.

### Other independent variables

Self-reported sex, chronological age and sites of residency were collected during the study visit. A composite score of cardiovascular health was calculated using information on the LS7 scale, namely smoking, physical activity, body mass index, total cholesterol, blood pressure, fasting plasma glucose and adherence to a Mediterranean-style diet^38^. The score ranged from 0 to 12, with higher numbers reflecting better cardiovascular health^38^.

### Outcome measure

Vital status was ascertained by linkage with the Registry office of the municipality of residency from the day after the baseline interview up to the end of January 2024.

### Statistics and reproducibility

We used Cox regression to examine the relationships between standardized values of epigenetic clocks and mortality across three models: ‘Baseline epigenetic clock only,’ ‘Change over time of epigenetic clock only’ and ‘Both baseline and change over time of epigenetic clock.’ All models were adjusted for baseline chronological age, sex and study site and subsequently further adjusted for the LS7 score. To examine whether the association with mortality was different among the relatively older and relative younger participants in the cohort (stratified by median age 68.68 years), we then included an interaction term between group (a binary variable indicating older versus younger than the median age) and baseline epigenetic ages. Results are displayed as forest plots, with C-statistics reported for all three models, and model fit assessed by likelihood ratio tests of nested models. HRs fail to provide a direct measure of how well biomarkers can classify risk, and C-statistics may not be sufficiently sensitive to detect meaningful improvements. Therefore, we also calculated the IDI and NRI, which have been proposed as more sensitive alternatives to evaluate improvement in risk prediction^39–41^. As more than 40% of participants were censored administratively at the end of follow-up, we used the R package ‘survIDINRI’ by Uno et al. to calculate IDI and NRI at 20 years follow-up with 1,000 bootstrap sampling. To estimate the age-specific rate of change in epigenetic clocks, we used a linear mixed model with random intercept and random slope, compared models using polynomial curves with different degrees of freedom and chose the model with the lowest Akaike information criterion. We conducted our analysis and plotting in SAS v.9.4 and R v.3.6.2, using the ‘survival’ package for survival analysis. All point estimates were reported with 95% CIs.

### Reporting summary

Further information on research design is available in the Nature Portfolio Reporting Summary linked to this article.

## Supplementary information

Supplementary Information Supplementary Tables 1–4 and Figs. 1–6.

Reporting Summary

Source data for Supplementary Figs. 1–6. Source Data Supplementary Fig. 1. Data for calculating the individual longitudinal rates of changes and plotting the correlation plot. Source Data Supplementary Fig. 2. Data for calculating the individual longitudinal rates of changes and plotting the correlation plot. Source Data Supplementary Fig. 3. Data for calculating the individual longitudinal rates of changes and plotting the correlation plot. Source Data Supplementary Fig. 4. Data for plotting the HR. Source Data Supplementary Fig. 5. Data for plotting the HR. Source Data Supplementary Fig. 6. Data for plotting the HR.

## Source data

Source Data Figs. 1–3. Source Data Fig. 1. Data for plotting the trajectories. Source Data Fig. 2. Data for plotting the HR. Source Data Fig. 3. Data for plotting the HR.

## Data Availability

The source data file from the InCHIANTI study can be requested by submitting a proposal to the committee following the instructions published at https://www.nia.nih.gov/inchianti-study. Source data are available with this paper.

## Code availability

Custom code was not developed specifically for this study.
