# Supplementary material for: Longitudinal changes in epigenetic clocks predict survival in the InCHIANTI cohort
Source: Nat Aging. 2026 Mar 13;6(3):534–40. doi: 10.1038/s43587-026-01066-6 (PMC13004684; doi:10.1038/s43587-026-01066-6)
Supplement: Supplementary file 1 — Supplementary Tables 1–4 and Figs. 1–6. [file 43587_2026_1066_MOESM1_ESM.pdf]

nature aging

Supplementary information

https://doi.org/10.1038/s43587-026-01066-6

Longitudinal changes in epigenetic clocks predict survival in the InCHIANTI cohort

In the format provided by the authors and unedited

Longitudinal changes in epigenetic clocks predict survival in the InCHIANTI cohort

Supplemental Table 1. Estimated Age-specific Annual Rate of Change in Epigenetic Clocks

Age	Estimated Annual Rate of Change in Epigenetic Clocks (year of epigenetic age per 1 year increase in chronological age)				
	Hannum	Horvath	DNAmPhenoAge	DNAmGrimAge (v1)	DNAmGrimAge-version 2
50	0.79 [0.76, 0.82]	0.82 [0.77, 0.86]	0.97 [0.93, 1.00]	0.65 [0.62, 0.68]	0.59 [0.57, 0.62]
55	0.79 [0.76, 0.82]	0.81 [0.77, 0.84]	0.97 [0.93, 1.00]	0.66 [0.63, 0.68]	0.61 [0.59, 0.64]
60	0.79 [0.76, 0.82]	0.80 [0.77, 0.83]	0.97 [0.93, 1.00]	0.67 [0.65, 0.69]	0.63 [0.61, 0.65]
65	0.79 [0.76, 0.82]	0.79 [0.76, 0.82]	0.97 [0.93, 1.00]	0.68 [0.66, 0.70]	0.65 [0.63, 0.66]
70	0.79 [0.76, 0.82]	0.78 [0.75, 0.81]	0.97 [0.93, 1.00]	0.69 [0.67, 0.71]	0.66 [0.64, 0.68]
75	0.79 [0.76, 0.82]	0.77 [0.74, 0.80]	0.97 [0.93, 1.00]	0.70 [0.68, 0.72]	0.68 [0.66, 0.70]
80	0.79 [0.76, 0.82]	0.76 [0.72, 0.79]	0.97 [0.93, 1.00]	0.71 [0.69, 0.73]	0.70 [0.67, 0.72]
85	0.79 [0.76, 0.82]	0.75 [0.71, 0.79]	0.97 [0.93, 1.00]	0.72 [0.69, 0.75]	0.71 [0.69, 0.74]
90	0.79 [0.76, 0.82]	0.74 [0.69, 0.79]	0.97 [0.93, 1.00]	0.73 [0.70, 0.76]	0.73 [0.70, 0.76]

Age	Estimated Annual Rate of Change in Epigenetic Clocks (change in “pace of aging” per 1 year increase in chronological age)	
	DunedinPOAm_38	DunedinPACE
50	-0.001210 [-0.002019, -0.000401]	0.002630 [0.001791, 0.003470]
55	-0.000928 [-0.001618, -0.000239]	0.003056 [0.002341, 0.003771]
60	-0.000646 [-0.001240, -0.000053]	0.003482 [0.002867, 0.004098]
65	-0.000365 [-0.000901, 0.000172]	0.003908 [0.003353, 0.004463]
70	-0.000083 [-0.000612, 0.000446]	0.004334 [0.003788, 0.004881]
75	0.000199 [-0.000375, 0.000773]	0.004760 [0.004168, 0.005352]
80	0.000481 [-0.000179, 0.001141]	0.005186 [0.004506, 0.005867]
85	0.000763 [-0.000011, 0.001537]	0.005612 [0.004814, 0.006410]
90	0.001045 [0.000139, 0.001950]	0.006038 [0.005104, 0.006972]

	Estimated Annual Rate of Change in Epigenetic Clocks (year of epigenetic age per 1 year increase in chronological age)			
Age	PCHannum	PCHorvath1	PCPhenoAge	PCGrimAge
50	0.58 [0.53, 0.63]	0.67 [0.64, 0.69]	0.74 [0.69, 0.78]	0.61 [0.59, 0.64]
55	0.60 [0.56, 0.64]	0.67 [0.64, 0.69]	0.75 [0.71, 0.80]	0.63 [0.61, 0.64]
60	0.62 [0.58, 0.66]	0.67 [0.64, 0.69]	0.77 [0.74, 0.81]	0.64 [0.62, 0.65]
65	0.64 [0.60, 0.67]	0.67 [0.64, 0.69]	0.79 [0.76, 0.82]	0.65 [0.64, 0.67]
70	0.65 [0.62, 0.69]	0.67 [0.64, 0.69]	0.81 [0.78, 0.84]	0.66 [0.65, 0.68]
75	0.67 [0.63, 0.71]	0.67 [0.64, 0.69]	0.83 [0.79, 0.86]	0.68 [0.66, 0.69]
80	0.69 [0.65, 0.73]	0.67 [0.64, 0.69]	0.84 [0.81, 0.88]	0.69 [0.67, 0.71]
85	0.70 [0.66, 0.75]	0.67 [0.64, 0.69]	0.86 [0.82, 0.91]	0.70 [0.68, 0.72]
90	0.72 [0.67, 0.78]	0.67 [0.64, 0.69]	0.88 [0.83, 0.93]	0.71 [0.69, 0.74]

Supplemental Table 2. Adjusted Hazard Ratios of Mortality for the protein proxy in GrimAge version 2 when both Baseline and Longitudinal Changes are included (N=699)

Variable Name	HR [95% CI]	Variable Name	HR [95% CI]
Baseline - DNAmadm	**1.32 [1.05 , 1.65]**	Longitudinal Changes - DNAmadm	1.07 [0.95 , 1.21]
Baseline - DNAmB2M	**1.45 [1.06 , 1.98]**	Longitudinal Changes - DNAmB2M	1.06 [0.94 , 1.20]
Baseline - DNAmCystatin_C	**1.64 [1.25 , 2.15]**	Longitudinal Changes - DNAmCystatin_C	**1.15 [1.02 , 1.29]**
Baseline - DNAmGDF_15	**1.72 [1.39 , 2.13]**	Longitudinal Changes - DNAmGDF_15	**1.09 [1.00 , 1.18]**
Baseline - DNAmleptin	0.94 [0.73 , 1.20]	Longitudinal Changes - DNAmleptin	0.93 [0.81 , 1.06]
Baseline - DNAmlogA1C	0.92 [0.81 , 1.05]	Longitudinal Changes - DNAmlogA1C	1.01 [0.90 , 1.13]
Baseline - DNAmlogCRP	**1.20 [1.06 , 1.35]**	Longitudinal Changes - DNAmlogCRP	**1.21 [1.08 , 1.36]**
Baseline - DNAmpai_1	0.98 [0.87 , 1.12]	Longitudinal Changes - DNAmpai_1	1.02 [0.91 , 1.15]
Baseline - DNAmTIMP_1	**2.13 [1.28 , 3.54]**	Longitudinal Changes - DNAmTIMP_1	**1.17 [1.05 , 1.31]**

Supplemental Table 3. C-statistics of models for epigenetic markers of aging (N=699)		
Survival Models (*)		
Hannum Clock	Concordance index	P-value (**)
Baseline only	0.7958	0.010
Slope only	0.7944	0.007
Both Baseline and Slope	0.7983	Ref
Horvath Clock		
Baseline only	0.7940	0.975
Slope only	0.7926	0.251
Both Baseline and Slope	0.7939	Ref
DNAmPhenoAge		
Baseline only	0.7978	<0.001
Slope only	0.7946	<0.001
Both Baseline and Slope	0.8006	Ref
DNAmGrimAge		
Baseline only	0.8039	0.019
Slope only	0.7928	<0.001
Both Baseline and Slope	0.8063	Ref
DNAmGrimAge version 2		
Baseline only	0.8039	0.003
Slope only	0.7942	<0.001
Both Baseline and Slope	0.8075	Ref
DunedinPOAm_38		
Baseline only	0.7961	0.029
Slope only	0.7931	<0.001
Both Baseline and Slope	0.7993	Ref
DunedinPACE		
Baseline only	0.7985	0.130
Slope only	0.7932	<0.001
Both Baseline and Slope	0.8004	Ref

(*) All the survival models include chronological age, sex, and site as covariates. For each epigenetic markers of aging, we fit three models (**"Baseline only"**, **"Slope only"**, and **"Both Baseline and Slope"**). **"Baseline only"** refers to the model including baseline epigenetic markers of aging, chronological age, sex, and study site. **"Slope only"** refers to the model including annual rate of longitudinal changes in epigenetic markers of aging, chronological age, sex, and study site. **"Both Baseline and Slope"** refers to the model including baseline epigenetic markers of aging, annual rate of longitudinal changes in epigenetic markers of aging, chronological age, sex, and study site.
(**) Because the **"Baseline only"** and **"Slope only"** models are nested in **"Both Baseline and Slope"** model, likelihood ratio test is used for model comparison with **"Both Baseline and Slope"** as the reference.

Supplemental Table 4. Integrated Discrimination Improvement and Net Reclassification Performance of Models including Epigenetic Markers of Aging (N=699)

Epigenetic Markers of Aging	IDI	NRI
Hannum Clock	**0.010 [0.000, 0.026]**	0.186 [-0.161, 0.300]
Horvath Clock	0.001 [-0.001, 0.009]	0.152 [-0.204, 0.237]
DNAmPhenoAge	**0.017 [0.005, 0.035]**	**0.251 [0.084, 0.320]**
DNAmGrimAge	**0.027 [0.010, 0.045]**	**0.236 [0.053, 0.316]**
DNAmGrimAge version 2	**0.029 [0.013, 0.051]**	**0.198 [0.003, 0.291]**
DunedinPOAm_38	0.009 [-0.001, 0.024]	0.126 [-0.087, 0.222]
DunedinPACE	**0.010 [0.001, 0.027]**	0.035 [-0.127, 0.158]

The IDI and NRI are used to assess the additional prediction values of models including epigenetic models of aging. Specifically, it compares the following two models for each epigenetic markers of aging: (i) model including chronological age, sex, and study site, and (ii) model including “baseline epigenetic markers of aging”, “annual rate of longitudinal changes in epigenetic markers of aging”, chronological age, sex, and study site.

IDI = Integrated Discrimination Improvement Index; NRI = net reclassification index

Supplemental Figure 1. Scatterplot of baseline epigenetic markers of aging and annual rate of longitudinal changes in the same epigenetic marker (N=699)

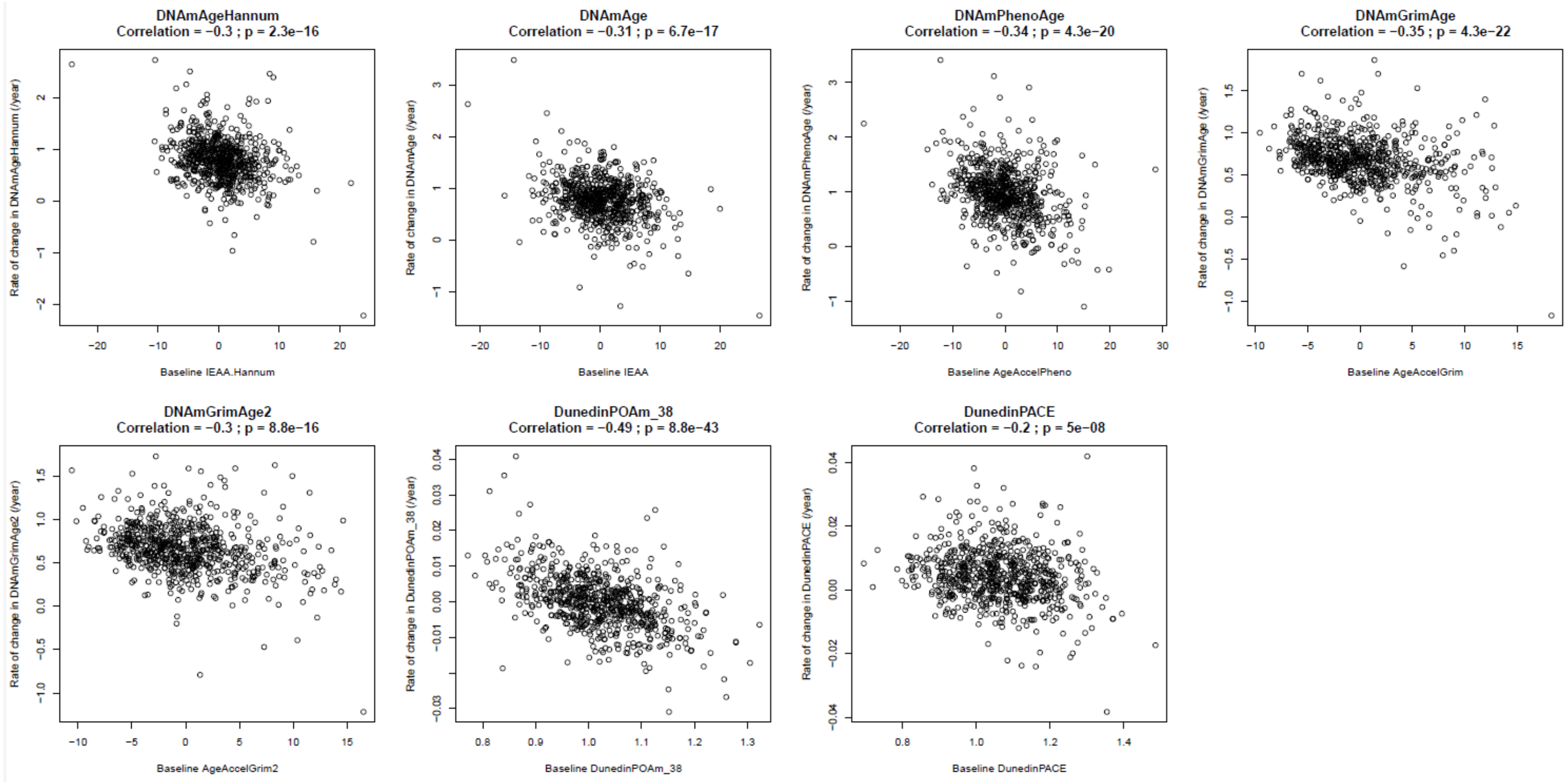

Because Hannum clock, Horvath clock, DNAmPhenoAge, DNAmGrimAge and DNAmGrimAge version 2 are highly correlated with chronological age, the chronological age adjusted variables are used for these measurements at the baseline. Specifically, IEAA.Hannum, IEAA, AgAccelPheno, AgeAccelGrim and AgeAccelGrim2 are used for Hannum clock, Horvath clock, DNAmGrimAge and DNAmGrimAge version 2 at the baseline, respectively.

Supplemental Figure 2. Correlations between epigenetic markers of aging at baseline and chronological age (N=699)

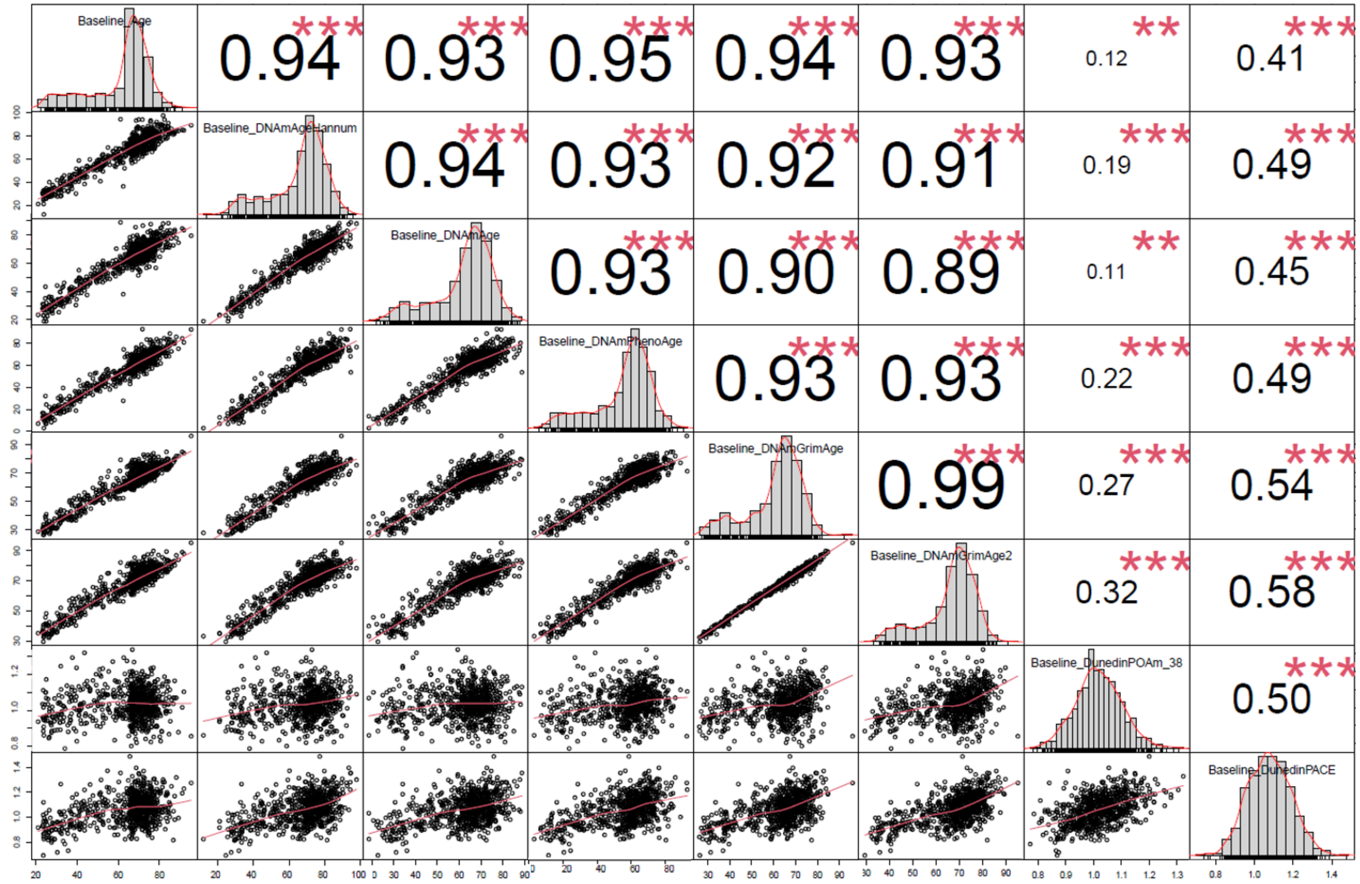

This figure showed that Hannum clock, Horvath clock, DNAmPhenoAge, DNAmGrimAge and DNAmGrimAge version 2 were all highly correlated with chronological age. Thus, chronological age adjusted variables are used for these measurements at the baseline in this analysis. Specifically, IEAA.Hannum, IEAA, AgAccelPheno, AgeAccelGrim and AgeAccelGrim2 are used for Hannum clock, Horvath clock, DNAmGrimAge and DNAmGrimAge version 2 at the baseline.

Supplemental Figure 3. Correlations between longitudinal changes in epigenetic markers of aging, and chronological age

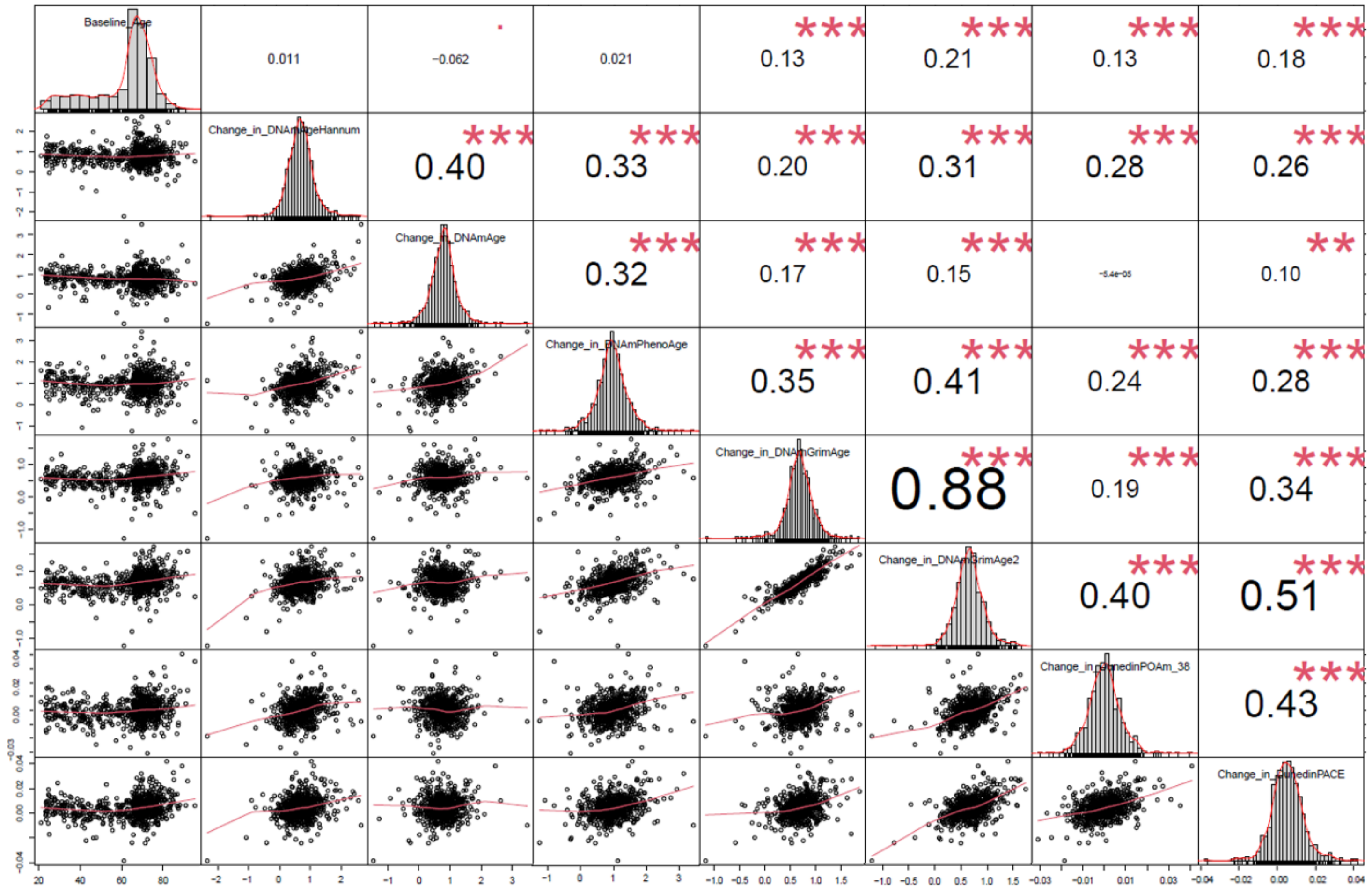

The Supplemental Figure 3 shows the correlation between baseline age and annual changes in epigenetic clocks. The diagonal shows the distribution of individual annual rate of changes in Hannum clock, Horvath clock, DNAmPhenoAge, DNAmGrimAge DNAmGrimAge version 2, Dunedin-POAm38 and DunedinPACE.

Supplemental Figure 4. Forest Plot for the Adjusted Hazard Ratios of Mortality when both Baseline and Longitudinal Changes are included in the model with additional adjustment for Life Simple Seven (N=674)

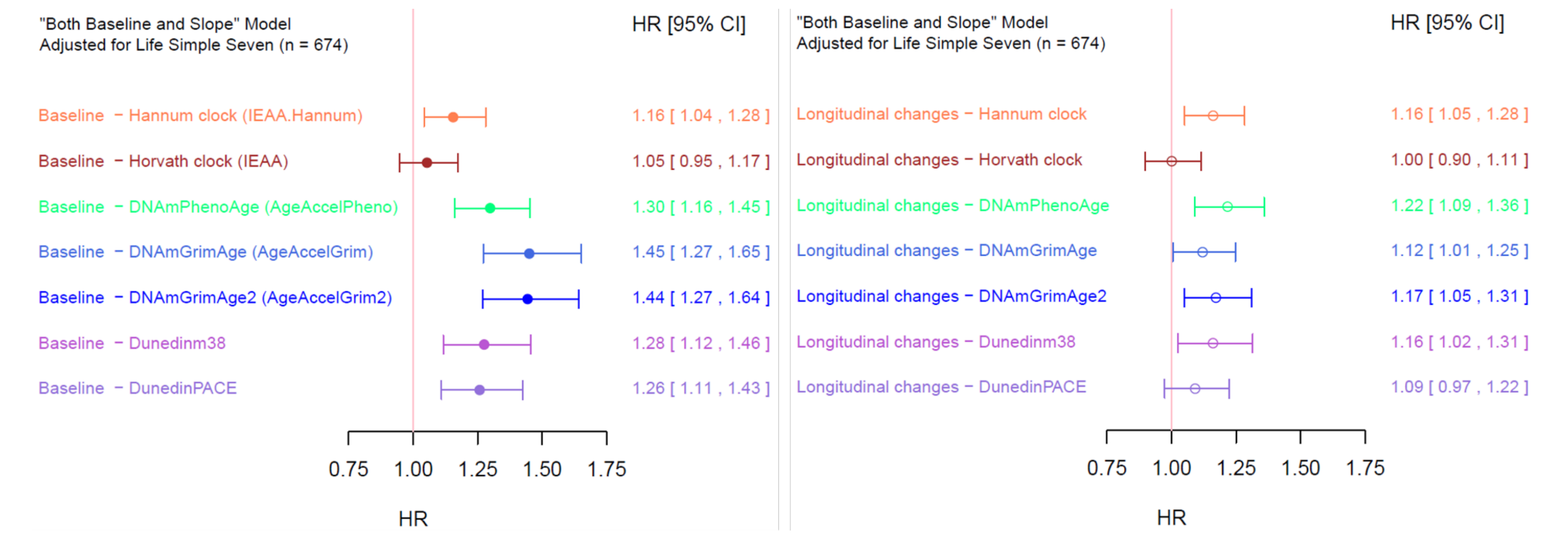

For Supplemental Figure 4, the hazard ratios are derived from the model including baseline epigenetic markers of aging, annual rate of longitudinal changes in epigenetic markers of aging, chronological age, sex, study site, and Life Simple Seven. Because Hannum clock, Horvath clock, DNAmPhenoAge and DNAmGrimAge are highly correlated with chronological age, chronological age adjusted variables are used for these measurements at the baseline. Specifically, IEAA.Hannum, IEAA, AgAccelPheno, AgeAccelGrim and AgeAccelGrim2 are used for Hannum clock, Horvath clock, DNAmPhenoAge, DNAmGrimAge and DNAmGrimAge version 2 at the baseline in the model, respectively. To facilitate the interpretation and comparison, all the coefficients are standardized.

Supplemental Figure 5. Forest Plot for the Adjusted Hazard Ratios of Mortality of PC clocks when both Baseline and Longitudinal Changes are included in the model (N=699)

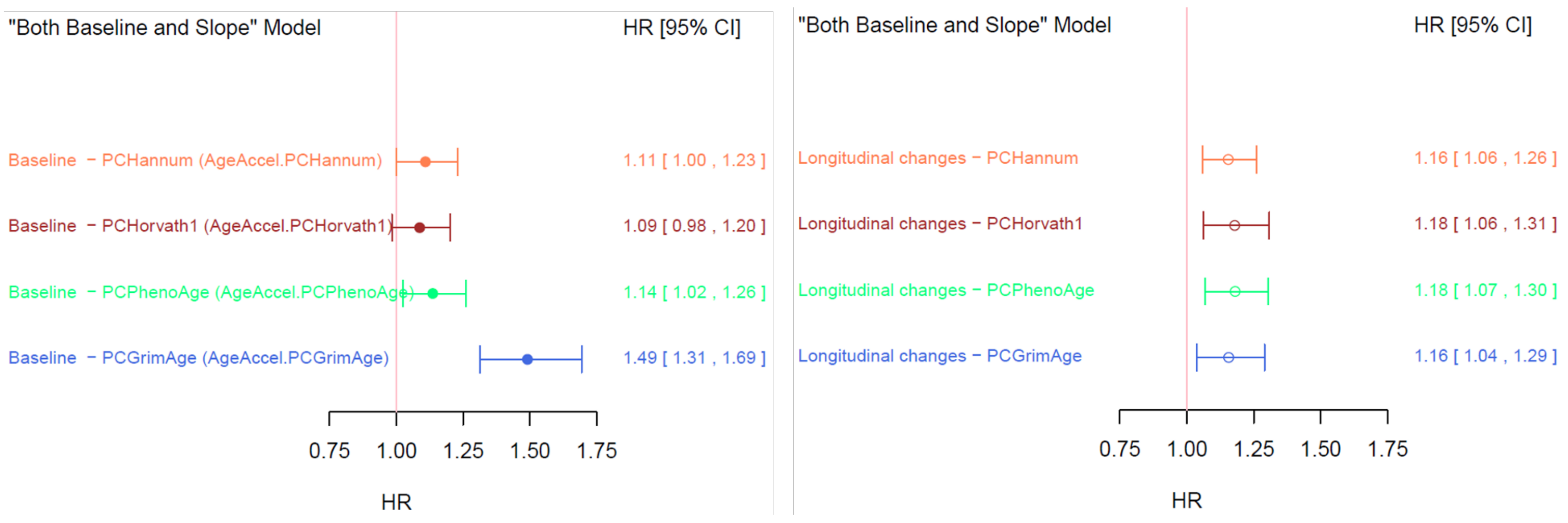

For Supplemental Figure 5, the hazard ratios are derived from the model including baseline age acceleration of PC clock, annual rate of longitudinal changes in PC clock, chronological age, sex, study site. Because these PC clocks are highly correlated with chronological age, the chronological age adjusted variables are used for these measurements at the baseline. Specifically, AgeAccel.PCHannum, AgeAccel.PCHorvath1, AgeAccel.PCPhenoAge, AgeAccel.PCGrimAge are used for PCHannum, PCHorvath1, PCPhenoAge, PCGrimAge at the baseline in the model, respectively. To facilitate the interpretation and comparison, all the coefficients are standardized.

Supplemental Figure 6. Forest Plot for the Adjusted Hazard Ratios of Mortality when comparing older group to the younger group

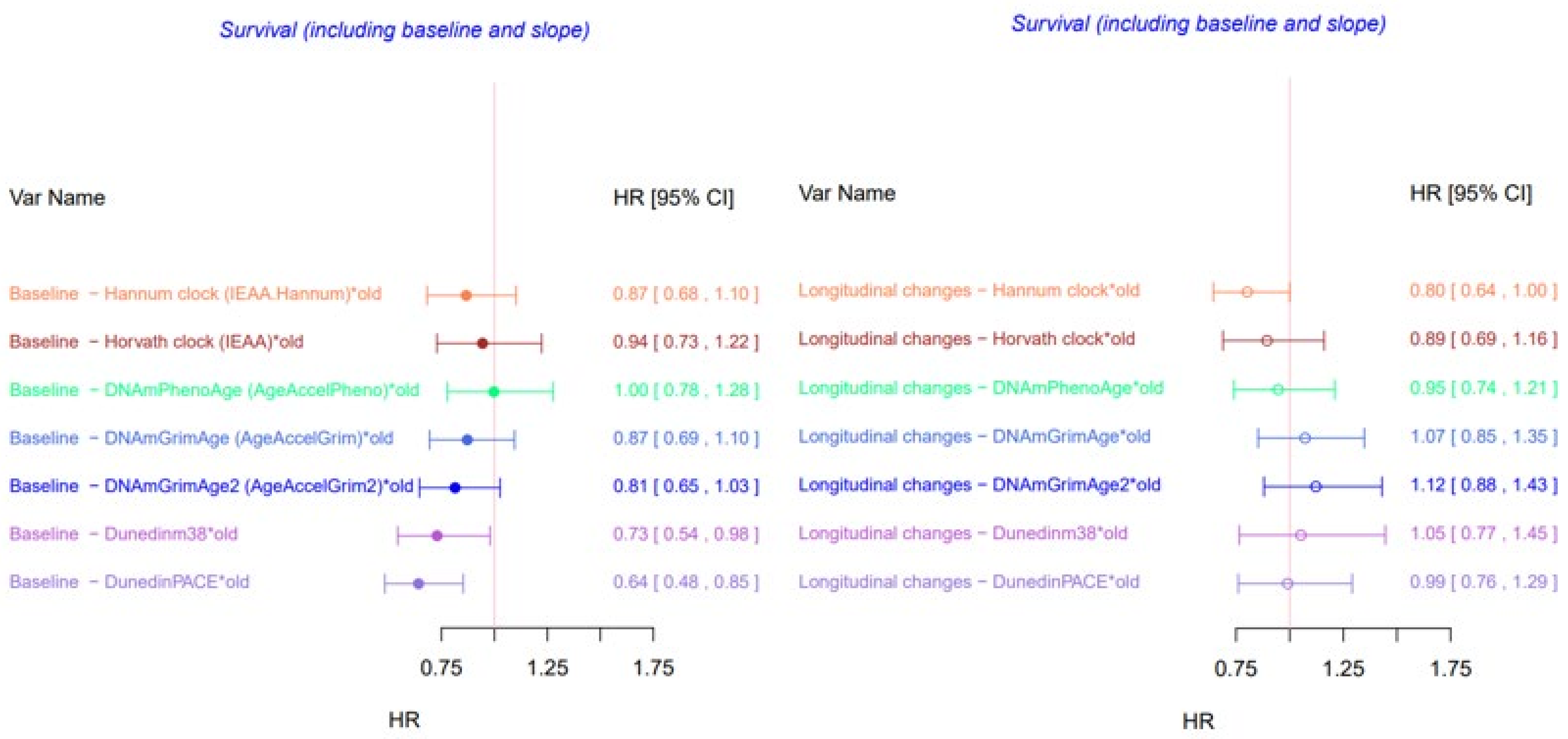

We further examined whether the association between the baseline epigenetic clock and mortality and the association between the longitudinal changes in the epigenetic clock differs by chronological age. Therefore, we created a binary indicator (old versus young using the median age as cutoff), and added two interactions, namely the interaction between the group indicator (old vs young) and baseline epigenetic clock and the group indicator (old vs young) and the longitudinal changes in epigenetic clock, in the model that includes both baseline and longitudinal changes in epigenetic clock. In this figure (Supplemental Figure 6), the hazard ratios are derived from the model additionally including the interaction between the group indicator (old vs young) and baseline epigenetic clock and the group indicator (old vs young) and the longitudinal changes in epigenetic clock. Therefore, a hazard ratio less than one implies the association with mortality attenuates in the older group when compared to the younger group.
